# Multi-modal assessment of neurovascular coupling during cerebral ischaemia and reperfusion using remote middle cerebral artery occlusion

**DOI:** 10.1177/0271678X16669512

**Published:** 2016-01-01

**Authors:** Brad A Sutherland, Jonas C Fordsmann, Chris Martin, Ain A Neuhaus, Brent M Witgen, Henning Piilgaard, Micael Lønstrup, Yvonne Couch, Nicola R Sibson, Martin Lauritzen, Alastair M Buchan

**Affiliations:** 1 Radcliffe Department of Medicine, University of Oxford, Oxford, UK; 2 School of Medicine, Faculty of Health, University of Tasmania, Hobart, Australia; 3 Department of Neuroscience and Pharmacology, University of Copenhagen, Copenhagen, Denmark; 4 Cancer Research UK and Medical Research Council Oxford Institute for Radiation Oncology, Department of Oncology, University of Oxford, Oxford, UK; 5 Department of Psychology, The University of Sheffield, Sheffield, UK; 6 Department of Clinical Neurophysiology, Glostrup Hospital, Glostrup, Denmark

**Keywords:** Neurovascular coupling, stroke, middle cerebral artery occlusion, cerebral blood flow, alteplase

## Abstract

Hyperacute changes in cerebral blood flow during cerebral ischaemia and reperfusion are important determinants of injury. Cerebral blood flow is regulated by neurovascular coupling, and disruption of neurovascular coupling contributes to brain plasticity and repair problems. However, it is unknown how neurovascular coupling is affected hyperacutely during cerebral ischaemia and reperfusion. We have developed a remote middle cerebral artery occlusion model in the rat, which enables multi-modal assessment of neurovascular coupling immediately prior to, during and immediately following reperfusion. Male Wistar rats were subjected to remote middle cerebral artery occlusion, where a long filament was advanced intraluminally through a guide cannula in the common carotid artery. Transcallosal stimulation evoked increases in blood flow, tissue oxygenation and neuronal activity, which were diminished by middle cerebral artery occlusion and partially restored during reperfusion. These evoked responses were not affected by administration of the thrombolytic alteplase at clinically used doses. Evoked cerebral blood flow responses were fully restored at 24 h post–middle cerebral artery occlusion indicating that neurovascular dysfunction was not sustained. These data show for the first time that the rat remote middle cerebral artery occlusion model coupled with transcallosal stimulation provides a novel method for continuous assessment of hyperacute neurovascular coupling changes during ischaemia and reperfusion, and offers unique insight into hyperacute ischaemic pathophysiology.

## Introduction

A wide variety of animal models have been used to understand the pathophysiology of ischaemic stroke as a means to identify biochemical events that can be targeted to prevent neuronal cell death. As a result, neuroprotective agents targeting the ischaemic cascade have been trialled, but, as has been extensively described, none of these have successfully translated to the clinic for ischaemic stroke.^1,2^ This lack of efficacy could be accounted for by methodological differences between the pre-clinical animal experiments and the clinical trials.^2^ One critical difference is the time of administration, with the hyperacute time points recognised as the critical periods for intervention to restrict brain injury following stroke but patient recruitment at this time can be limited. In addition, physiological factors such as cerebral blood flow (CBF) and body temperature could account for the translational failure of neuroprotective agents for stroke.^3^

Reduced CBF is an important mediator of cerebral ischaemic injury. Animal models of both transient and permanent middle cerebral artery occlusion (MCAO) have demonstrated that the extent of CBF reduction and restoration of CBF upon reperfusion is inversely correlated with infarct volume, i.e. the greater the CBF, the lesser the infarct.^4,5^ The regulation of CBF post-ischaemia is therefore crucial for recovery, and understanding the pathological milieu associated with changes in CBF immediately after the onset of ischaemia is critical in the development of adjunct therapies for stroke.

The regulation of CBF is tightly controlled and influenced by a number of mediators released from neurons and astrocytes, which act directly or indirectly on vascular smooth muscle cells and pericytes.^6–8^ Interestingly, a number of mediators that control CBF also play an important role within the ischaemic cascade, which leads to neuronal cell death, including glutamate, intracellular Ca^2+^ increase, prostaglandin formation and nitric oxide formation.^1,7,8^ This link between the molecular determinants of ischaemic stroke pathogenesis and CBF control can lead to neurovascular deficits,^9^ which may be associated with impaired neuronal connectivity, plasticity and repair.^10–12^ It is thought that neurovascular dysfunction can persist for days and is dependent on the level of the ischaemic insult.^8^ However, it is not known how neurovascular coupling is affected during ischaemia and reperfusion in the hyperacute setting. Such knowledge may shed light on new opportunities for pharmacological intervention to modulate hyperacute neurovascular effects.

Recombinant tissue plasminogen activator (rtPA; administered as the formulation alteplase) is the only approved thrombolytic treatment for ischaemic stroke, but only a small proportion of patients receive benefit.^13^ It is usually administered as a 10% intravenous bolus and the remaining 90% infused over 60 min. It has been shown that endogenous tPA plays a role in neurovascular coupling through the nitric oxide pathway.^14^ However, it is unknown how alteplase, independent of its thrombolytic effects, alters neurovascular coupling during the first hour of reperfusion following MCAO.

The aims of this study, therefore, were threefold. First, to use a remote method for inducing MCAO that enables continuous and uninterrupted (i.e. before, during and after occlusion) assessment of neurovascular function in vivo using a range of neurophysiological and neuroimaging techniques. Second, to establish how alteplase administration affects neurovascular function upon reperfusion following MCAO. Third, to compare the results obtained under aim one with those of a ‘conventional’ MCAO approach with measurement of neurovascular function 24 h following transient occlusion–reperfusion. This approach will for the first time provide a novel method for continuous assessment of hyperacute neurovascular coupling changes during ischaemia and reperfusion, and will be a crucial tool for investigating hyperacute pathophysiological changes in the ischaemic brain.

## Methods

### Animals

All procedures conformed to the Animal (Scientific Procedures) Act 1986 (UK) and the European Council’s Convention for the Protection of Vertebrate Animals Used for Experimental and Other Scientific Purposes, and were approved by the University of Oxford Animal Ethics Committee, the Home Office (UK) and the Danish National Ethics Committee. Experiments are reported in line with the ARRIVE guidelines. Male Wistar rats (241–340 g, n = 26; Harlan, UK or Charles River, Denmark) were used. All animals were housed in a 12 h light/dark cycle and had ad libitum access to food and water prior to experiments.

Three cohorts of animals were used (see Supplementary Figure 1 for flow of rats through the study). In the first cohort (remote MCAO), the acute effects of occlusion–reperfusion on neurovascular coupling were assessed under a single episode of non-recovery general anaesthesia. The second cohort (remote MCAO + alteplase) was assessed for the neurovascular effects of occlusion–reperfusion + alteplase or vehicle(s) infusion, again under a single episode of non-recovery general anaesthesia. The third cohort (90 min MCAO induced in the conventional way^5 ^+ 22.5 h reperfusion) was used to determine the effects of transient MCAO upon neurovascular coupling 22.5 h after occlusion–reperfusion.

### Remote MCAO preparation

The remote MCAO model is a modification of the conventional intraluminal filament MCAO model previously described.^5^ This model allows the advancement of the filament to occlude the MCA while the rat is secured in a stereotaxic frame, and permits the assessment of CBF and electrophysiological responses at very acute time points following occlusion or filament retraction. Anaesthesia was induced with 4% isoflurane and maintained with 1.5–2% isoflurane in 30–50% oxygen and 70–50% nitrogen. Initially, the right femoral artery was cannulated with polyethylene tubing (ID 0.4 mm, OD 0.8 mm) for blood gas monitoring and the right femoral vein was cannulated using the same size tubing for administration of drugs or alpha-chloralose anaesthesia as described below. After the cannulations, the MCAO preparatory procedure was carried out on the right side of the rat. A midline incision was made in the neck, and the right external carotid artery permanently ligated. The right common carotid artery was permanently ligated and the internal carotid and pterygopalatine arteries were isolated and temporarily ligated. An arteriotomy was made in the common carotid artery, and a guide cannula (polyethylene tubing ID 0.4 mm, OD 0.8 mm, length 3–4 cm shorter than the length of the filament; Smiths Medical (Ashford, UK)) was inserted into the common carotid artery up to the beginning of the internal carotid artery. A custom-length 4-0 filament (10–15 cm, Supplementary Fig 2A) with a silicon tip (0.37 mm diameter, 5–6 mm in length; Doccol, Sharon, MA, USA) was advanced through the guide cannula into the internal carotid artery. The filament continued to be advanced up the internal carotid artery past the pterygopalatine branch until the silicon could no longer be observed, at which point the filament was secured (Supplementary Fig 2B). At this point, the filament did not occlude the MCA, but a minor advancement of the filament will be sufficient to produce an occlusion. Following this, the rat was tracheotomised and ventilated to enable breathing rate and tidal volume to be altered to maintain blood gas values within physiological ranges throughout the experiment (pH 7.35–7.5, pCO_2_ 30–45 mmHg, pO_2_ 90–150 mmHg). Core body temperature was maintained (37℃) throughout the procedure using a homeothermic heating pad attached to a rectal probe. Following preparatory surgery for remote MCAO, animals were turned over and placed into a stereotaxic frame, with the filament and guide cannula remaining accessible. Each rat then underwent preparation for assessment of neurovascular coupling.

### Neurovascular coupling assessment

After placement of the animal in a stereotaxic frame, a midline incision of the scalp was made and the skin retracted. Two equal 4 mm × 4 mm craniotomies with a centre point at 3 mm posterior and 4 mm lateral to bregma were drilled over the right (ipsilateral to ischaemia) and left (contralateral to ischaemia) somatosensory cortex, which is a region that is supplied by the MCA. In some cases, only small burr holes were drilled on the skull over each hemisphere, rather than craniotomies. Craniotomies were superfused with artificial CSF (composition in mM): 120 NaCl, 2.8 KCl, 22 NaHCO_3_, 1.45 CaCl, 1.0 Na_2_HPO_4_ and 0.876 MgCl_2_, pH 7.43, heated to 37℃ and bubbled with 5% CO_2_, 21% O_2_, 74% atmospheric air. MK-801 (1 mM) was applied topically to the contralateral craniotomy to prevent seizure activity and spreading depression.^15^ Following completion of surgical procedures, anaesthesia was switched to α-chloralose HBC complex in saline initiated by i.v. bolus of 0.8 g/kg and maintained at 0.55 g/kg/h i.v. using an infusion pump. Either Laser Speckle Contrast Imaging (LSCI) of both hemispheres (Moor Instruments, Axminster, UK) or Laser Doppler Flowmetry (LDF) of the ipsilateral (right) ischaemic hemisphere only (Perimed, Järfälla, Sweden) was used to assess CBF on the surface of the brain, whilst a modified Clark-type polarographic oxygen microelectrode (OX-10, Unisense) was used to monitor tpO_2_ at approximate coordinates of 3 mm posterior and 6 mm lateral to Bregma, at a depth of 300–600 µm from the ipsilateral cortical surface (see Fordsmann et al.^16^ for further details). For some experiments, a glass microelectrode to record extracellular local field potentials was also placed in the ipsilateral cortex at a depth of 300–600 µm to assess neuronal activity.^16^ Transcallosal stimulation (10 Hz, 0.2 ms pulse width, 15–16 s duration, 2 mA, 1 or 2 min inter-stimulation interval repeated for 3–10 trials) was initiated through a bipolar stimulating electrode (Kopf Instruments, Tujunga, CA, USA) placed in the left craniotomy/burr hole so its tip was 300–600 µm below the surface of the brain. This stimulation evoked a response in CBF, tpO_2_ and neuronal activity in the right somatosensory cortex (ipsilateral to MCAO, contralateral to stimulation), which was recorded as described above (Supplementary Fig 2C). The region of activation with transcallosal stimulation as described above has previously been shown to evoke responses in the somatosensory barrel cortex in a similar manner to infraorbital nerve stimulation, which innervates the whisker pad.^17^

### Cohort 1: Remote MCAO

**Figure 1.**
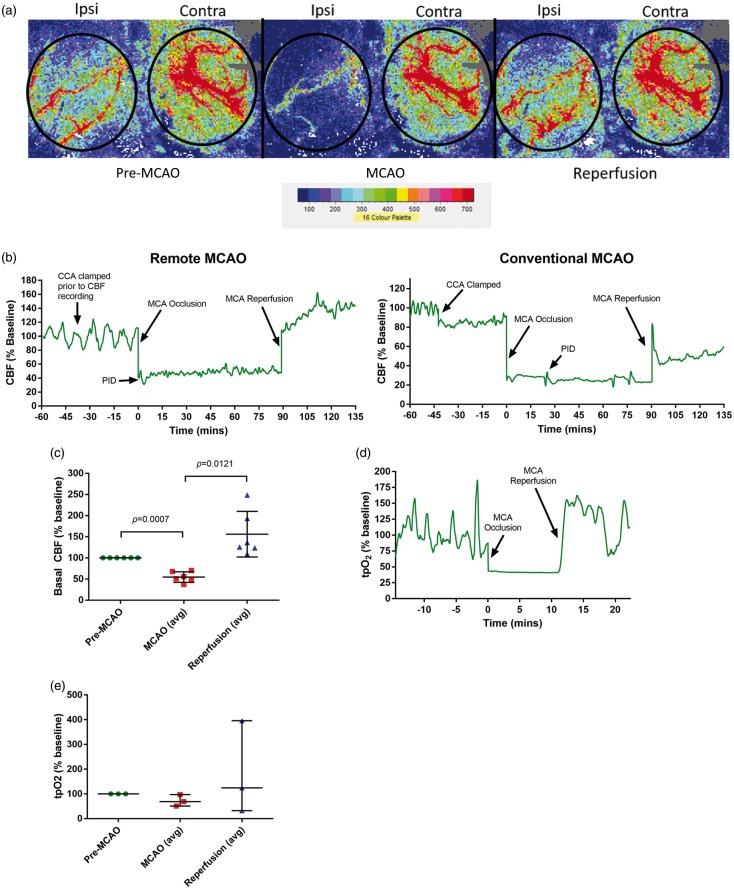
Remote MCAO produces changes in resting CBF and tissue oxygenation. (a) Example Laser Speckle Contrast images of the superficial cortex. Remote MCAO produces a significant drop in blood flow upon filament advancement over the ipsilateral cortex. Filament retraction leads to a large increase in CBF throughout the ipsilateral cortex. Note that even pre-stroke, CBF is reduced in the ipsilateral cortex compared with the contralateral cortex due to prior clamping of the CCA. (b) Remote MCAO: example CBF trace of the induction of ischaemia with the remote MCAO model and restoration of CBF upon reperfusion. Note that CCA was clamped prior to recording and so 100% CBF baseline is post-CCAO (approximately 65% of true baseline). Conventional MCAO: example CBF trace of the induction of ischaemia with the conventional MCAO model and restoration of CBF upon reperfusion while keeping the CCA ligated. (c) Resting CBF (averaged over each period; mean ± SD) revealing the effects of remote MCAO and reperfusion on CBF after CCAO had already occurred. N = 6 per group. (d) Example trace of tpO_2_ following induction of ischaemia with the remote MCAO model and restoration of tpO_2_ upon reperfusion. (e) Resting tpO_2_ (averaged over each period; median ± range) revealing the effects of remote MCAO and reperfusion on tissue oxygenation. N = 3 per group.

After preparation for remote MCAO and neurovascular coupling (see above), 11 rats had remote MCAO induced by gently advancing the filament through the guide cannula until a drop in CBF could be observed using LSCI (Figure 1(a)) or LDF (Figure 1(b)). The filament remained in place for a pre-determined period of time and was retracted to produce reperfusion, as evidenced by a sharp increase in CBF (Figure 1(a) and (b)). Transcallosal or whisker stimulation and evoked response assessment as described above were carried out prior to MCAO (but after common carotid artery ligation, when CBF has partially been reduced), during MCAO and during reperfusion. All remote MCAO experiments (cohort 1 + cohort 2, n = 20) were conducted under one non-recovery general anaesthesia session.

### Cohort 2: Remote MCAO + alteplase

We used the remote MCAO model to assess whether alteplase administration at the time of reperfusion altered neurovascular coupling acutely. The remote MCAO model and neurovascular coupling experiments were carried out on the stereotaxic frame as described above (n = 9). Remote MCAO duration was 90 min, which is a timepoint within the limits for alteplase treatment and provides a moderate level of brain infarction.^18^ During remote MCAO, transcallosal stimulations (10 Hz, 0.2 ms pulsewidth, 15 s duration, 3 mA, 2 min inter-stimulation interval repeated for at least three trials) occurred at 1, 30, 60 and 80 min into MCAO. Reperfusion was produced through filament retraction, and saline (n = 3), carrier (containing 31.5 mg/kg L-arginine, 9 mg/kg phosphoric acid and 0.1 mg/kg polysorbate 80; n = 3) or alteplase (containing 0.9 mg/kg r-tPA and the carrier (Boehringer Ingelheim, Ingelheim, Germany); n = 3) was immediately administered with 10% of the dose as i.v. bolus and 90% of the dose as an i.v. infusion lasting 1 h in line with human stroke treatment. Animals were randomly assigned to treatment group following confirmation of successful ischaemia and the researcher was blind to the treatment group that was administered. Transcallosal stimulations occurred at 3, 30 and 50 min into the reperfusion period. CBF, tpO_2_ and local field potentials were monitored throughout the reperfusion period. The timepoints for transcallosal stimulation were chosen to obtain neurovascular assessment as soon as possible after ischaemia and reperfusion, while keeping evenly spaced (every 30 min) assessment throughout both periods. The last stimulation occurred 10 min before the end of each period due to the duration of the stimulation protocol.

### Cohort 3: Conventional MCAO with recovery

To determine whether any acute changes in neurovascular coupling were sustained, six animals were randomly assigned to undergo either 90 min MCAO using the conventional method or sham with a 22.5 h recovery period, at which time neurovascular coupling assessment was carried out. Conventional MCAO was achieved through introduction of the filament into the external carotid artery, which was advanced to occlude the MCA for 90 min as previously described.^5^ Sham animals had the filament inserted up to the pterygopalatine branch of the internal carotid artery, but no further. The filament was retracted and the common carotid artery ligation was removed for reperfusion. LDF (Oxford Optronix, Oxford, UK) was used to determine successful ischaemia and reperfusion.^5,19^ Each animal was then allowed to recover from anaesthesia and placed in a recovery cage with regular monitoring until neurovascular coupling assessment. After 22.5 h of reperfusion, animals were assessed for neurological deficit using the modified Garcia neuroscore.^5,20^ Animals were then re-anaesthetised, tracheotomised and ventilated, the femoral artery was cannulated for blood gas monitoring (all blood gases were within the limits described above) and the skull over the ipsilateral somatosensory cortex (same 4 mm × 4 mm area) thinned for LSCI. Animals underwent neurovascular coupling assessment as described above, with the following exceptions: stimulation electrodes (Grass Technologies, Warwick, RI, USA) were placed in the contralateral whisker pad of the animal to evoke a response in the ipsilateral whisker barrel cortex in the same region as transcallosal stimulation; 1.25% isoflurane carried in 30% O_2_ and 70% N_2_ was used; no tpO_2_ measurements took place; whisker pad was stimulated at either 3 Hz or 10 Hz, 0.3 ms pulsewidth, 16 s duration, 2 mA, 1 min inter-stimulation interval repeated for 10 trials. Animals were then killed, the brain dissected out, sliced into 2 mm thick sections and stained for infarct location using 2,3,5-triphenyltetrazolium chloride (TTC), as described previously.^5^

### Data analysis

LSCI CBF data were acquired using Moor FLPI software (Moor Instruments), and exported to Matlab (Mathworks, Natick, MA, USA) to determine changes in both resting and evoked CBF over time. LDF CBF, tPO_2_ data and local field potential recordings were obtained using Spike software (Cambridge Electrical Design, Cambridge, UK) and analysed using Matlab. Evoked responses were calculated as the amplitude of the peak or the area under the curve of the entire response. The cerebral metabolic rate for O_2_ (CMRO_2_) was calculated using the same method as Fordsmann et al.^16^ All resting CBF, tpO_2_ and CMRO_2_ data had a running average of 1 min applied before analysis of changes in response to remote MCAO. All evoked CBF, tpO_2_ and CMRO_2_ data had a running average of 1 s applied before analysis of changes in response to transcallosal stimulation as previously described.^16^ The running average analysis method was used to smooth stochastic noise (see Supplementary Fig 3). Data are presented as mean ± standard deviation (SD) or median ± range and are annotated in the figure legends. Repeated measures ANOVA followed by Tukey’s post-hoc test was used to compare groups or Dunnet’s post-hoc test when comparing to pre-MCAO group only. For correlation analysis, a Pearson correlation coefficient was determined between two variables. *p* < 0.05 was considered statistically significant. All statistical tests were two-sided and no further adjustment for multiple comparison has been made for the overall number of tests. Specific n numbers are stated for each experiment in the results or figures. No power calculation for sample size was used as a new model was being developed and no estimate of effect size could be established.

## Results

### Cohort 1: Diminished evoked responses after remote MCAO

Initially, we aimed to determine how the remote MCAO model of stroke with the rat positioned on a stereotaxic frame performed compared with the conventional method of MCAO by studying a number of different parameters.^5^ Specifically, we assessed resting CBF and tpO_2_ changes immediately following remote MCAO and reperfusion. The LSCI and LDF data showed that CBF was diminished upon advancement of the filament to occlude the MCA (Suppl Movie 1), and was restored by retracting the filament (Figure 1(a) and (b), Suppl Movie 2). Quantification of CBF minima revealed that the remote MCAO model reduced CBF in the superficial cortex to 36.0% ± 14.4% (n = 6) of baseline (Tukey’s: *p* = 0.0003; Supplementary Fig 4A). Average CBF over the entire ischaemic period showed that remote MCAO decreased CBF to 54.6% ± 12.6% (n = 6) of baseline (Tukey’s: *p* = 0.0007; Figure 1(c)). Note that CBF recording with the remote MCAO model could only begin following common carotid artery occlusion (CCAO) and filament placement (see methods, Figure 1(b)), and that CCAO CBF reduction using the conventional MCAO method has previously been reported by us to be 64.6% ± 11.9% (n = 44) of baseline.^5^ Therefore, transforming the CBF levels during remote MCAO to include the extra drop by CCAO would equate to a true relative minimum CBF of 23.2% ± 9.3% and average CBF of 35.3% ± 8.1%. Retraction of the filament increased maximum CBF to 210% ± 91% (Tukey’s: *p* = 0.0135, n = 6; Supplementary Fig 4A) and average CBF to 156% ± 54% (Tukey’s: *p* = 0.0121, n = 6; Figure 1(c)) of post-CCAO baseline showing that the acute hyperemia upon reperfusion occurs in a similar manner as with conventional MCAO. In addition to CBF, the remote MCAO model also produced a decrease in tpO_2_ upon MCAO (median 69% (range 51–97%) of baseline throughout MCAO period, only n = 3 due to technical problems with electrode) that was maintained throughout the duration of ischaemia, and could be restored upon reperfusion (Figure 1(d) and (e), Supplementary Fig 4B).

Even though MK-801 was applied topically to the contralateral cortex to prevent spreading depolarization due to electrical stimulation, we could also observe peri-infarct depolarizations (PIDs) in the ischaemic hemisphere during remote MCAO (Figure 1(b)). PIDs occurred in 10/15 (67%) animals during remote MCAO where it was assessed. Over all experiments using the remote MCAO model, we had a success rate of 18 out of 20 animals (90%) as assessed by a drop in CBF by at least 30% of baseline (which equates to a drop to 45% of true baseline when the CBF reduction by CCAO is considered). The reasons for failure of remote MCAO included resistance of the filament to advancing remotely (one animal) and a loose guide cannula (one animal) (see Supplementary Fig 1).

**Figure 2.**
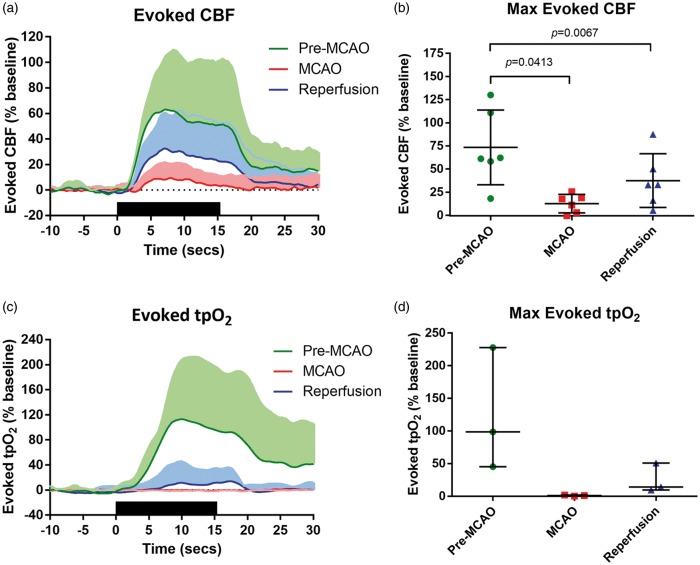
Remote MCAO diminishes evoked CBF and tissue oxygen, which was partially reversed by reperfusion. (a) Mean + SD trace of the evoked CBF response to transcallosal stimulation pre-, during and post-remote MCAO. (b) Quantification (mean ± SD) of the maximum change in CBF in response to transcallosal stimulation pre-, during and post-remote MCAO. N = 6 per group. (c) Mean + SD trace of the evoked tpO_2_ response to transcallosal stimulation pre-, during and post-remote MCAO. (d) Quantification (median ± range) of the maximum change in tpO_2_ in response to transcallosal stimulation pre-, during and post-remote MCAO. N = 3 per group.

**Figure 3.**
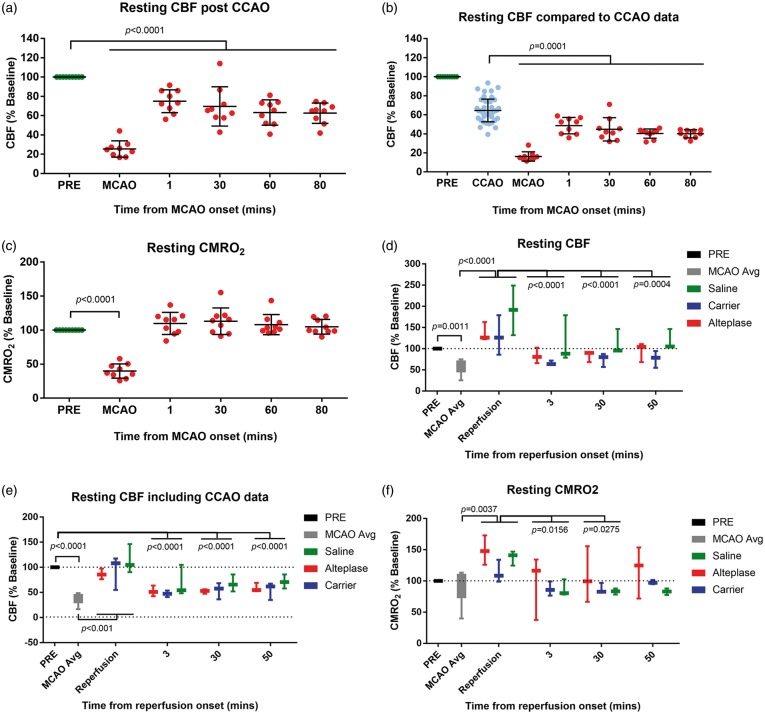
Remote MCAO changes in resting CBF and oxygen metabolism were not affected by alteplase. (a) Resting CBF dropped with remote MCAO, and then partially rebounded, but CBF remained attenuated compared with PRE-MCAO throughout the ischaemic period. Note that PRE was recorded after CCA occlusion. (b) Conventional MCAO data show common carotid artery occlusion (CCAO) drops CBF to 65% of baseline (n = 44)^5^ and so CBF values from (a) were transformed to obtain % CBF change with respect to pre-CCAO. (c) Resting CMRO_2_ dropped at the point of remote MCAO but was restored to pre-MCAO levels throughout the ischaemic period. (d) Resting CBF increased upon filament retraction following remote MCAO with no significant changes between any treatment groups during the reperfusion period. These values were normalised to pre-MCAO after CCAO. (e) Same as (d) except values were normalised to pre-MCAO before CCAO. Apart from the initial reperfusion, the resting CBF remained significantly dampened throughout the reperfusion period and this was not affected by alteplase. (f) Resting CMRO_2_ increased upon filament retraction following remote MCAO, but then decreased back to baseline levels throughout the reperfusion period. Resting CMRO_2_ was not affected by alteplase. (a–c) had N = 9 at each time point and is presented as mean ± SD. (d–f) had N = 3 per group at each time point and is presented as median ± range.

The remote MCAO model was chosen to carry out evoked blood flow and oxygen experiments immediately prior to occlusion, throughout the ischaemic period, and immediately following reperfusion without having to move the animal. Using the transcallosal stimulation model (Supplementary Fig 2C) prior to MCAO, we observed strong evoked CBF responses (73% ± 40% (n = 6) of basal CBF) lasting the duration of the stimulation in both somatosensory cortices (Figure 2(a) shows only right cortex (ipsilateral to MCAO) CBF, Suppl Movie 3). However, upon induction of remote MCAO where resting CBF drops in the ipsilateral cortex, the maxima of evoked CBF responses were significantly reduced to 13% ± 10% (Tukey’s: *p* = 0.0413, n = 6) of basal CBF (Figure 2(a) and (b)). When reperfusion was restored by filament retraction, evoked CBF responses partially recovered to 37% ± 29% (n = 6) of basal CBF (Figure 3(a) and (b)) but were still significantly decreased compared with baseline when measured as maxima (Tukey’s: *p* = 0.0067, Figure 2(b)) and area (Tukey’s: *p* = 0.0359, Supplementary Fig 4C). Similar changes in evoked tpO_2_ were also observed, whereby a strong transient increase in tpO_2_ upon stimulation (99% (45–228%), n = 3) was diminished following remote MCAO (1.1% (0.2–2.1%), n = 3), and was partially restored upon reperfusion (14% (10–51%), n = 3; Figure 2(c) and (d)). Similar results were observed when area of tpO_2_ was measured following stimulation (Supplementary Fig 4D). These results suggest that MCAO can lead to abrupt attenuation of the evoked CBF and oxygen response, which could be partially recovered upon reperfusion.

### Cohort 2: Diminished evoked responses after remote MCAO in the presence of alteplase

One advantage of the remote MCAO model is the ability to investigate neurovascular coupling hyperacutely in the presence or absence of vasoactive drugs. Endogenous tPA has been shown to modulate neurovascular coupling,^14^ and so we assessed the effects of alteplase on resting CBF and neurovascular coupling immediately upon reperfusion following remote MCAO. Alteplase, saline or L-arginine vehicle was infused immediately upon filament withdrawal. In this cohort of animals, remote MCAO induction produced a sudden drop in resting CBF that partially rebounded, but ischaemia was maintained throughout the MCAO period (Tukey’s: all MCAO timepoints compared with pre-MCAO had *p* < 0.0001, n = 9; Figure 3(a)). When the pre-existing CCAO-induced CBF drop was taken into account, CBF remained attenuated throughout the ischaemic period (Tukey’s: all MCAO timepoints (n = 9) compared with CCAO (n = 44) had *p* < 0.0001; Figure 3(b)). Resting CMRO_2_ at the point of occlusion was decreased (Tukey’s: *p* < 0.0001, n = 9), but was soon restored to pre-MCAO levels and maintained at this level throughout the ischaemic period (Figure 3(c)). During remote MCAO, all animals (n = 9) had at least one PID (mean = 3 PIDs, range 1–6) with the average time to first PID being 3.9 min (range 2–7 min).

Remote MCAO was followed by successful reperfusion after retraction of the filament (Tukey’s: *p* < 0.0001, n = 9; Figure 3(d) and (e)), while alteplase did not influence CBF throughout the reperfusion period (Figure 3(d)). When normalising to pre-CCAO values, resting CBF remained lower (Tukey’s: *p* < 0.0001, n = 9) than baseline throughout the reperfusion period, which was not affected by treatment group (Figure 3(e)). An increase in resting CMRO_2_ was also observed upon reperfusion (Tukey’s: *p* = 0.0037; n = 9) but recovered quickly to baseline, and this was not affected by treatment group (Figure 3(f)). Only one animal out of all cohort two animals (11%) during the 60 min reperfusion period presented a PID, which occurred at 45 min following reperfusion onset.

**Figure 4.**
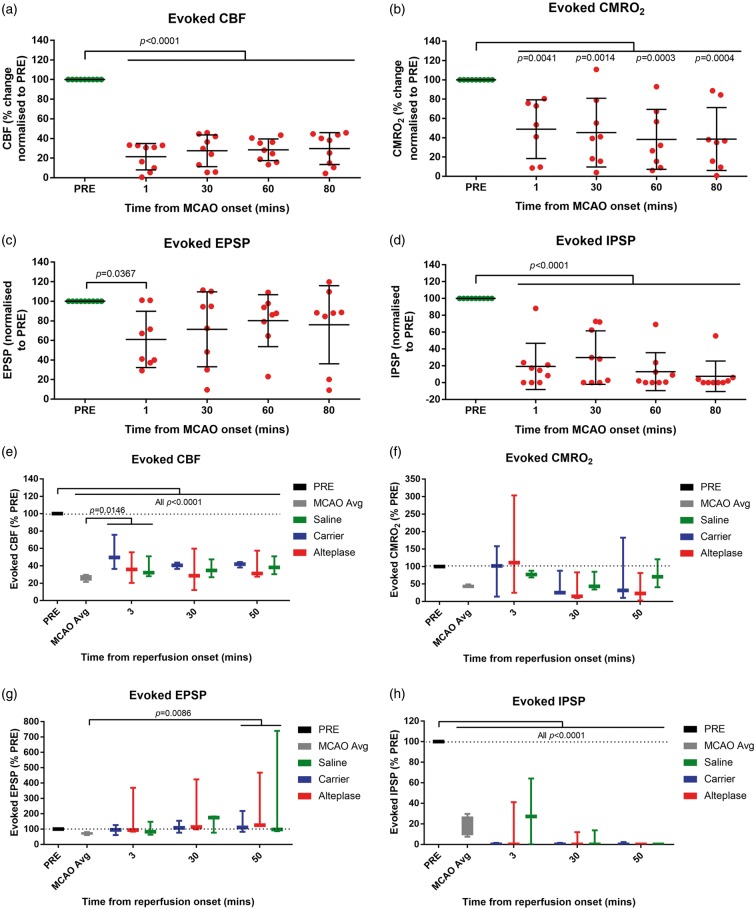
Remote MCAO changes in evoked CBF, oxygen metabolism and neuronal activity were not affected by alteplase. (a) Remote MCAO decreased evoked CBF in response to transcallosal stimulation. (b) Remote MCAO diminished evoked CMRO_2_. (c) Evoked EPSP was reduced initially following remote MCAO. (d) Remote MCAO diminished evoked IPSP. (e) Evoked CBF in response to transcallosal stimulation during reperfusion was slightly increased compared with MCAO, attenuated compared with PRE-MCAO but was not affected by alteplase. (f) Reperfusion and alteplase treatment did not affect evoked CMRO_2_. (g) Evoked EPSP increased at 50 min of reperfusion but was not affected by alteplase. (h) Evoked IPSP remained abolished upon reperfusion in all treatment groups. (a–d) had N = 9 at each time point and presented as mean ± SD. (e–h) had N = 3 per group at each time point and presented as median ± range. All values were normalised to evoked changes pre-MCAO.

Using the same transcallosal stimulation model as described above, evoked CBF during remote MCAO was diminished at all time points during the 90 min ischaemic period (Tukey’s: *p* < 0.0001; Figure 4(a) (15 s stim CBF maxima), Supplementary Fig 5B (15 s stim CBF area), Supplementary Fig 5C (2 s stim CBF area)). Similar attenuation of evoked CMRO_2_ was also observed during MCAO (Tukey’s: *p* < 0.0042, n = 9; Figure 4(b)). An electrode placed into the somatosensory cortex to measure excitatory post-synaptic potential (EPSP) and inhibitory post-synaptic potential (IPSP; Suppl Fig 5A) revealed that there was a small reduction in summed EPSP response upon remote MCAO (Tukey’s: *p* = 0.0367, n = 9; Figure 4(c)) that was not maintained throughout the ischaemic period while IPSP was abolished upon remote MCAO (Tukey’s: *p* < 0.0001, n = 9; Figure 4(d)).

Reperfusion partially recovered evoked CBF responses (Tukey’s: *p* = 0.0146, n = 9) but remained dampened throughout reperfusion (Tukey’s: *p* < 0.0001, n = 9; Figure 4(d)). Reperfusion did not alter evoked CMRO_2_ (Figure 4(f)). At 50 min of reperfusion, evoked EPSP was augmented (Tukey’s: *p* = 0.0086, n = 9; Figure 4(g)) while IPSP remained attenuated after reperfusion (Tukey’s: *p* < 0.0001, n = 9; Figure 4(h)). Treatment with alteplase, L-arginine carrier or saline did not affect any of these parameters throughout the reperfusion period (Figure 4(e)–(h), Suppl Figs 5D, E) suggesting that alteplase does not produce any acute effects on neurovascular coupling.

**Figure 5.**
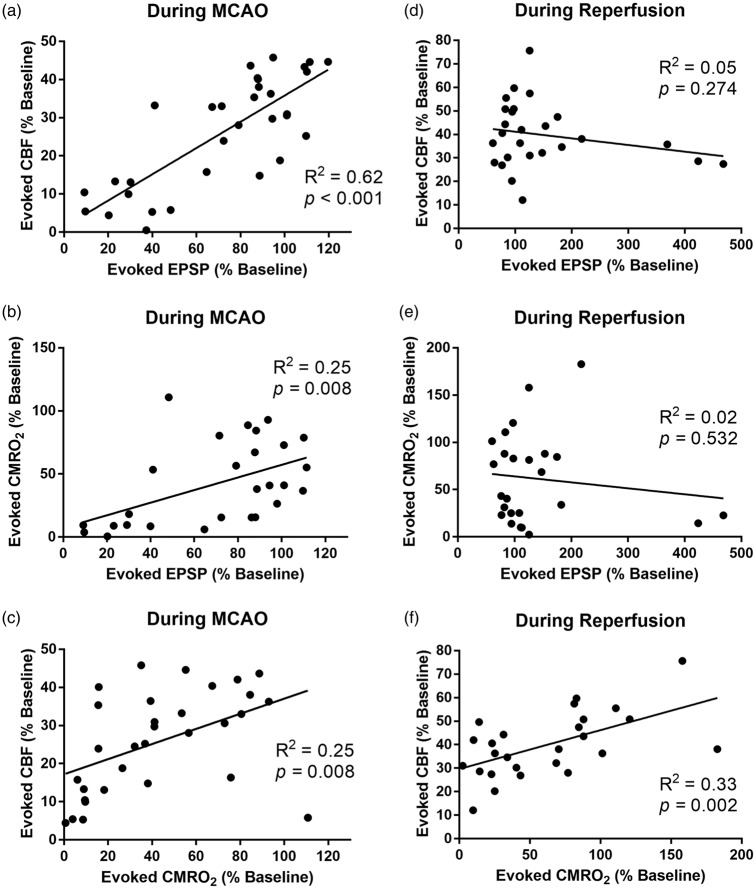
Correlations between evoked CBF, neuronal activity and metabolism during ischaemia and reperfusion. (a) There was a significant correlation between evoked CBF and neuronal activity during ischaemia. (b) There was a significant correlation between evoked CMRO_2_ and neuronal activity during ischaemia. (c) A significant correlation between evoked CBF and oxygen metabolism was observed during remote MCAO. (d) There was no significant correlation between evoked CBF and neuronal activity during reperfusion. (e) There was no correlation between evoked CMRO_2_ and neuronal activity during reperfusion after remote MCAO. (f) There was a significant correlation between evoked CBF and oxygen metabolism during reperfusion.

Further to this, correlation analyses revealed that during ischaemia evoked neuronal activity was significantly diminished and this correlated with the decrease in evoked CBF (Pearsons r^2 ^= 0.62, *p* < 0.001; Figure 5(a)) and evoked CMRO_2_ (Pearsons r^2 ^= 0.25, *p* = 0.008; Figure 5(b)). This evoked CBF decrease also correlated with a significant decrease in evoked CMRO_2_ (Pearsons r^2 ^= 0.22, *p* = 0.008; Figure 5(c)). This suggests that reduced resting CBF by remote MCAO leads to diminished evoked neuronal activity, which subsequently attenuates the evoked blood flow and metabolic responses. There was no significant correlation between the extent of evoked CBF response and resting CBF during ischaemia (Pearsons r^2 ^= 0.11, *p* = 0.053; Suppl Fig 6A).

During reperfusion, whilst there was a recovery of evoked neuronal activity, this was not correlated with an increase in evoked CBF (Pearsons r^2 ^= 0.05, *p* = 0.274; Figure 5(d)) and CMRO_2_ (Pearsons r^2 ^= 0.02, *p* = 0.532; Figure 5(e)) suggestive of a partial uncoupling of neuronal, hemodynamic and metabolic responses (breakdown of neurovascular coupling). However, evoked metabolism correlated with evoked CBF during reperfusion (Pearsons r^2 ^= 0.33, *p* = 0.002; Figure 5(f)) indicating that uncoupling occurred between the neuronal and hemodynamic responses. Interestingly, evoked CBF during reperfusion was also inversely correlated with levels of resting CBF during ischaemia (Pearsons r^2 ^= 0.57, *p* = 0.019; Suppl Fig 6B) and reperfusion (Pearsons r^2 ^= 0.25, *p* = 0.008; Suppl Fig 6C), whereby the lower the resting level of CBF, the higher the evoked CBF during reperfusion.

### Cohort 3: No diminished evoked responses 24 h following conventional MCAO

**Figure 6.**
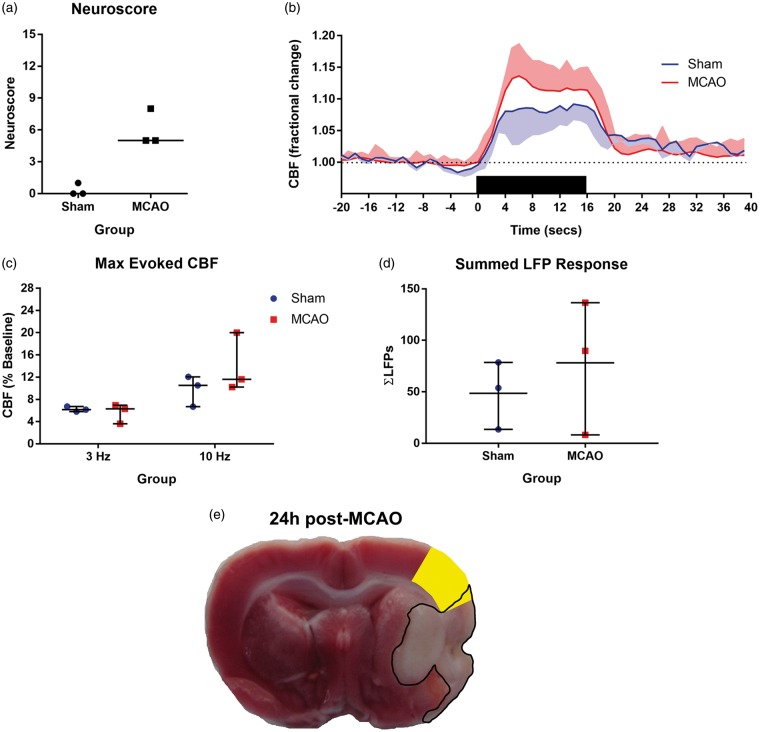
Neurovascular coupling is not impaired 24 h following 90 min MCAO. (a) Median neuroscore 24 h following 90 min MCAO or sham. MCAO animals had greater neurological deficit than sham animals. (b) Mean ± SD trace of evoked CBF in response to 10 Hz whisker stimulation 24 h following sham or 90 min MCAO. N = 3 per group. (c) Maximal CBF (median ± range) in response to 3 or 10 Hz whisker stimulation was unaltered at 24 h between sham and MCAO groups. (d) Neuronal activity following 3 Hz whisker stimulation measured by the summed local field potential response (median ± range) was not different between sham and MCAO groups at 24 h. (e) Representative TTC image of a brain section 24 h following 90 min MCAO showing the location of infarction (white with black outline) with an overlay (yellow) of the somatosensory cortex that had an increase in CBF evoked by whisker stimulation.

The remote MCAO model reduced evoked blood flow and metabolic responses during ischaemia and impaired neurovascular coupling within the first hour of reperfusion. Since neurovascular coupling changes might have been sustained beyond this, we used the whisker stimulation model to assess neurovascular coupling in animals exposed to the conventional 90 min MCAO model^5^ followed by recovery for 22.5 h. Sham controls were used to compare the neurovascular coupling effects of MCAO. Median neurological deficit was greater 24 h after MCAO onset compared with sham animals (Sham = 0 (range 0–1), MCAO = 5 (range 5–8), n = 3, Figure 6(a)). The whisker stimulation model (10 Hz) produced a strong evoked CBF response that lasted for the duration of the stimulation (Figure 6(b)). In contrast with the hyperacute reperfusion time points where evoked CBF was only partially restored, neurovascular coupling appears to recover by 24 h as there were no differences in either evoked CBF responses or local field potentials between sham and MCAO animals in the ischaemic hemisphere (Figure 6(b)–(d)). The transcallosal stimulation model at these frequencies activates pyramidal neurons within the somatosensory cortex,^21^ which notably was not in the infarcted area at 24 h post-MCAO but in the peri-infarct area that surrounds the core infarct (Figure 6(e)).

## Discussion

In this study, we used a remote intraluminal filament MCAO model of stroke to assess neurovascular coupling prior to, during and after ischaemia. This model is a crucial tool for studying hyperacute changes in CBF and has the ability to assess the effects of adjunct therapy on neurovascular function in the early stages after a stroke. The remote MCAO model leads to changes in CBF and brain oxygenation consistent with the conventional intraluminal filament MCAO model.^22^ We showed here that evoked CBF responses were diminished during ischaemia compared with pre-MCAO, alongside attenuated evoked tpO_2_, CMRO_2_ and neuronal activity. Upon reperfusion, while evoked excitatory neuronal activity recovered, there was only partial restoration of evoked CBF and CMRO_2_ indicative of disrupted neurovascular coupling. Furthermore, since these hyperacute time points are recognised as the critical periods for intervention in the clinical setting, the remote MCAO model also allowed investigation into the effects of vasoactive drugs on neurovascular coupling. In this study, we showed that the thrombolytic alteplase did not affect the neurovascular coupling response upon reperfusion when administered at a clinically relevant dose and route. The hyperacute impairment in neurovascular coupling during the first 60 min of reperfusion was not sustained at 22.5 h following 90 min conventional MCAO, and so neurovascular recovery of the peri-infarct tissue took place during this time. Overall, the ability to assess neurovascular coupling immediately upon ischaemia and reperfusion using the remote MCAO model will provide unique insight into the pathophysiology of ischaemic brain injury and changes in CBF and metabolism.

Neurovascular deficits following stroke have previously been reported in both animal^9–12^ and human studies,^23^ and these deficits may be associated with damaged neuronal connectivity and plasticity and lead to problems related to brain repair. Most studies have investigated later time points and showed that whilst there were somatosensory deficits in the days following stroke that can recover during the following weeks,^24^ at 10 weeks post-stroke there were functional connectivity impairments that can contribute to long-term deficits in somatosensory function.^12^ Our investigation into the hyperacute neurovascular effects revealed significant changes in the evoked responses upon transcallosal stimulation throughout ischaemia. In particular, both evoked excitatory and inhibitory neuronal activity was attenuated, which led to a decrease in evoked CBF, tpO_2_ and CMRO_2_ responses. However, upon reperfusion, evoked inhibitory neuronal activity was diminished whilst evoked excitatory neuronal activity was enhanced above baseline leading to its overactivation. This was associated with attenuated evoked CBF and CMRO_2_ responses, and correlation analyses suggest that there was impairment in the link between recovery of evoked neuronal activity and evoked hemodynamic and metabolic responses during reperfusion. Further experiments using the conventional MCAO model showed that this impaired neurovascular coupling response was not present by 24 h following MCAO onset. Stimulation at the frequencies used activates neurons only within the somatosensory cortex,^21^ which is located in the peri-infarct region outside the ischaemic core at 24 h following 90 min MCAO. These findings suggest that during ischaemia, neurovascular coupling remains intact but overall activity is downregulated; in the hyperacute phase (first hour of reperfusion) neurovascular coupling is impaired, which is followed by recovery in the acute phase (first 24 h of reperfusion).

In both cohorts 1 and 2, there was only a small recovery of evoked CBF responses upon transcallosal stimulation during reperfusion, even in the presence of excessive excitatory neuronal activity. This suggests that there is some impairment in neurovascular coupling during this time. It is currently unknown how long this impairment will persist for, but its sustained nature is surprising given the restoration of oxygen and glucose that accompanies reperfusion. One reason could be that during ischaemia brain cells conserve energy due to limited supply, but upon restoration of blood flow, the cellular machinery to produce energy and subsequent signalling from neurons and glia to the vasculature may remain impaired.^8^ Another contributor may be that in this model, the CCA remains ligated after filament retraction, meaning that full reperfusion does not occur, which could account for attenuated neurovascular coupling. Additional studies are needed to test these hypotheses.

In 67% of animals assessed, PIDs occurred during remote MCAO with only one animal from all cohorts (7%) exhibiting a PID during 60 min of reperfusion. Spreading depolarizations have long been reported to impair vascular reactivity in the cortex.^25–27^ A recent study has suggested that during ischaemia, somatosensory stimulation itself can lead to a transient worsening of the supply–demand mismatch within the brain, triggering a PID.^28^ The total period of depolarization contributes to ischaemic injury,^29^ and so it is conceivable that the neurovascular impairment seen in the present study is partially related to the occurrence of PIDs, in addition to ischaemia-reperfusion injury.

Alteplase, a thrombolytic, is administered to ischaemic stroke patients to restore blood flow, but only a minority of patients benefit from this therapeutic strategy.^13^ Given that endogenous tPA plays a role in neurovascular coupling through the nitric oxide pathway,^14^ we hypothesised that exogenous rtPA, given as alteplase, may alter neurovascular coupling during the first hour of reperfusion following MCAO. Since alteplase contains high levels of L-arginine, the substrate of nitric oxide production, and L-arginine has been speculated to fuel some of the non-thrombolytic effects of alteplase,^30^ we also assessed the effect of the vehicle containing L-arginine on resting CBF and neurovascular coupling following MCAO. In our hands, neither alteplase nor its L-arginine carrier altered resting CBF and CMRO_2_, evoked neuronal activity, CBF and CMRO_2_ upon stimulation during the first hour of reperfusion. This suggests that alteplase has no effect on the neurovascular coupling response during reperfusion, and that any effect on resting CBF remain solely limited to its thrombolytic activity. This is in line with a recent study that showed that alteplase had no effect on CBF immediately after conventional intraluminal filament MCAO nor neurotoxicity 24 h later.^5^

One advantage of the remote MCAO model is the ability to examine neurovascular coupling using a range of neuroimaging techniques prior to occlusion and immediately after ischaemia and reperfusion have occurred. This approach allows each animal to act as its own control and is important given most neurovascular coupling studies using animal models of stroke are unable to obtain baseline measurements prior to ischaemia, necessitating the use of sham animals to compare evoked changes. This can be observed with cohort 3 where sham controls were used rather than a pre-MCAO control, which could account for differences in evoked responses between the remote MCAO and conventional MCAO models.

We appreciate that there are a number of limitations to this study. The invasive carotid surgery, tracheotomy and ventilation for control of physiological parameters, and craniotomies meant that recovery experiments were not an option. Hence, the period of investigation was limited to 1 h of reperfusion following 90 min MCAO. Continuous CBF measurements could only be taken following CCAO, so normalising to CBF post-CCAO does not reflect true CBF. The extent of CBF drop upon CCAO can influence outcome following MCAO,^31^ and so it is important that CBF monitoring occurs upon CCAO in future studies. Upon filament insertion remotely, there was a sharp decrease in CBF indicating ischaemia had occurred but this was followed rapidly by a partial rebound of CBF (this was particularly evident in cohort 2 with LSCI). This rebound has been observed previously^32^ and could be due to recruitment of collateral vessels to the ischaemic area as an attempt to restore CBF.

The remote MCAO model on the stereotaxic frame has also been developed independently in the mouse not for neurovascular coupling assessment but to investigate the effects of ischaemia and reperfusion on oxygenation in the brain.^33^ Previous studies have successfully attempted the remote MCAO model in the magnet to investigate acute changes in MRI parameters following ischaemia.^34^ However, our personal experience was that the remote MCAO model in the magnet was very difficult to reliably reproduce due to the distance (approximately 100 cm) between the indwelling monofilament and access to the guide cannula to control filament position. If the remote MCAO model could be reproducibly developed for MRI studies, this will be a powerful technique to assess the dynamic changes in brain function and metabolism following stroke. Nevertheless, our study and the recently published mouse study, advocate the remote MCAO model as a powerful tool on the bench to examine the hyperacute effects of MCAO on blood flow, oxygenation, neuronal activity and metabolism in the brain.

In conclusion, the hyperacute effects of ischaemia and reperfusion on neurovascular coupling can be investigated using a remote MCAO model. Significant changes to evoked responses were observed during ischaemia, which only partially recovered during the initial stages of reperfusion. In particular, the evoked neuronal activity upon stimulation did not lead to pronounced evoked CBF and metabolic responses throughout the first 60 min of reperfusion, and these responses were not affected by the administration of the thrombolytic alteplase. However, the hyperacute diminished responses were restored by 22.5 h of reperfusion using the conventional MCAO model. This study provides insight into brain activity changes initiated upon ischaemia and the time-dependent recovery of neurovascular responses following reperfusion. Further studies that are able to relate hyperacute modulation of neurovascular coupling following MCAO to longer term tissue, neurovascular imaging or even behavioural outcomes will be useful for developing new hyperacute ‘neurovascular’ (as opposed to principally vascular or CBF-mediated) interventions that could protect the brain after a stroke.

## Supplementary Material

Supplementary material

Supplementary material

Supplementary material

Supplementary material
