# Supplementary material for: Multi-modal assessment of neurovascular coupling during cerebral ischaemia and reperfusion using remote middle cerebral artery occlusion
Source: J Cereb Blood Flow Metab. 2016 Jan 1;37(7):2494–508. doi: 10.1177/0271678X16669512 (PMC5531347; doi:10.1177/0271678X16669512)
Supplement: Supplementary material [file SupplementaryMaterial_9512.pdf]

Multi-modal assessement of neurovascular coupling during cerebral ischemia and reperfusion using remote middle cerebral artery occlusion

Brad A Sutherland[1,2], Jonas C Fordsmann[3], Chris Martin[4,5], Ain A Neuhaus[1], Brent M Witgen[3], Henning Piilgaard[3], Micael Lønstrup[3], Yvonne Couch[1], Nicola R Sibson[4], Martin Lauritzen[3,6,*], Alastair M Buchan[1,*]

Supplementary Information

Supplementary Methods

Alteplase experiments

For the alteplase experiments, at each timepoint we evoked responses with transcallosal stimulation using a 10 Hz, 0.2 ms pulsewidth, 15 sec duration, 3 mA, 2 min inter-stimulation interval paradigm repeated for 3 trials. In addition, we also evoked responses using 10 Hz, 0.2 ms pulsewidth, 2 sec duration, 3 mA, 30 sec inter-stimulation interval repeated for 5 trials (Supplementary Figs 5C, E) which showed the same results as the 15 sec stimulation (Supplementary Figs 5B, D). For these comparisons, evoked CBF responses were assessed using an area under the curve calculation.

Supplementary Figures

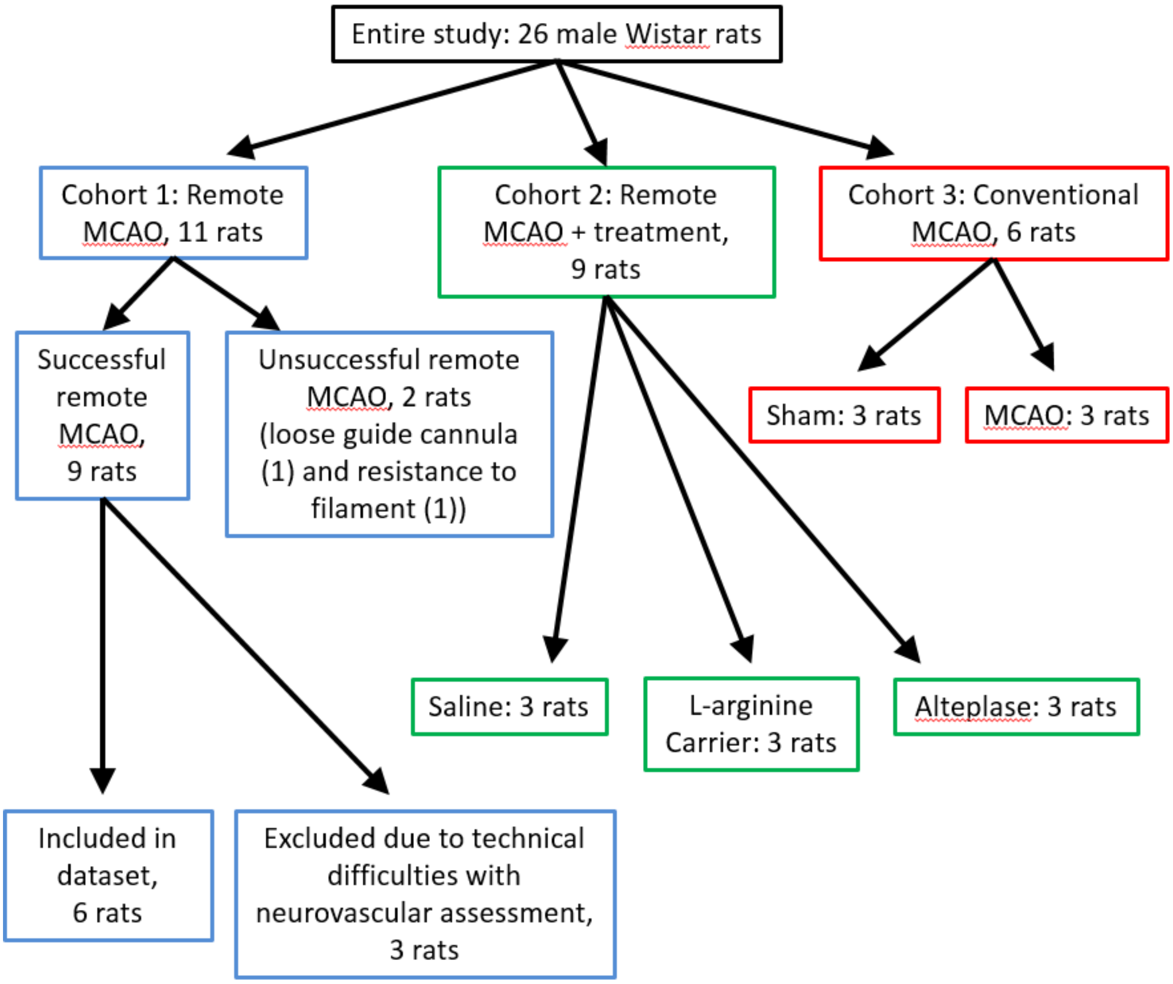

Supplementary Figure 1. Flow chart of animals in study. Overall, there were 26 male Wistar rats included in the study. 11 of these rats were included in cohort 1 to establish the remote MCAO model. Of these, 9 rats (82%) achieved successful remote MCAO but only 6 were presented in the dataset due to technical problems with the neurovascular assessment with 3 rats. Nine rats were included in cohort 2 to further confirm remote MCAO effects on neurovascular coupling and assess any effects of the thrombolytic alteplase. Of these, 9 rats (100%) achieved successful remote MCAO. Six rats were included in cohort 3 to determine neurovascular coupling effects 24h following conventional transient MCAO.

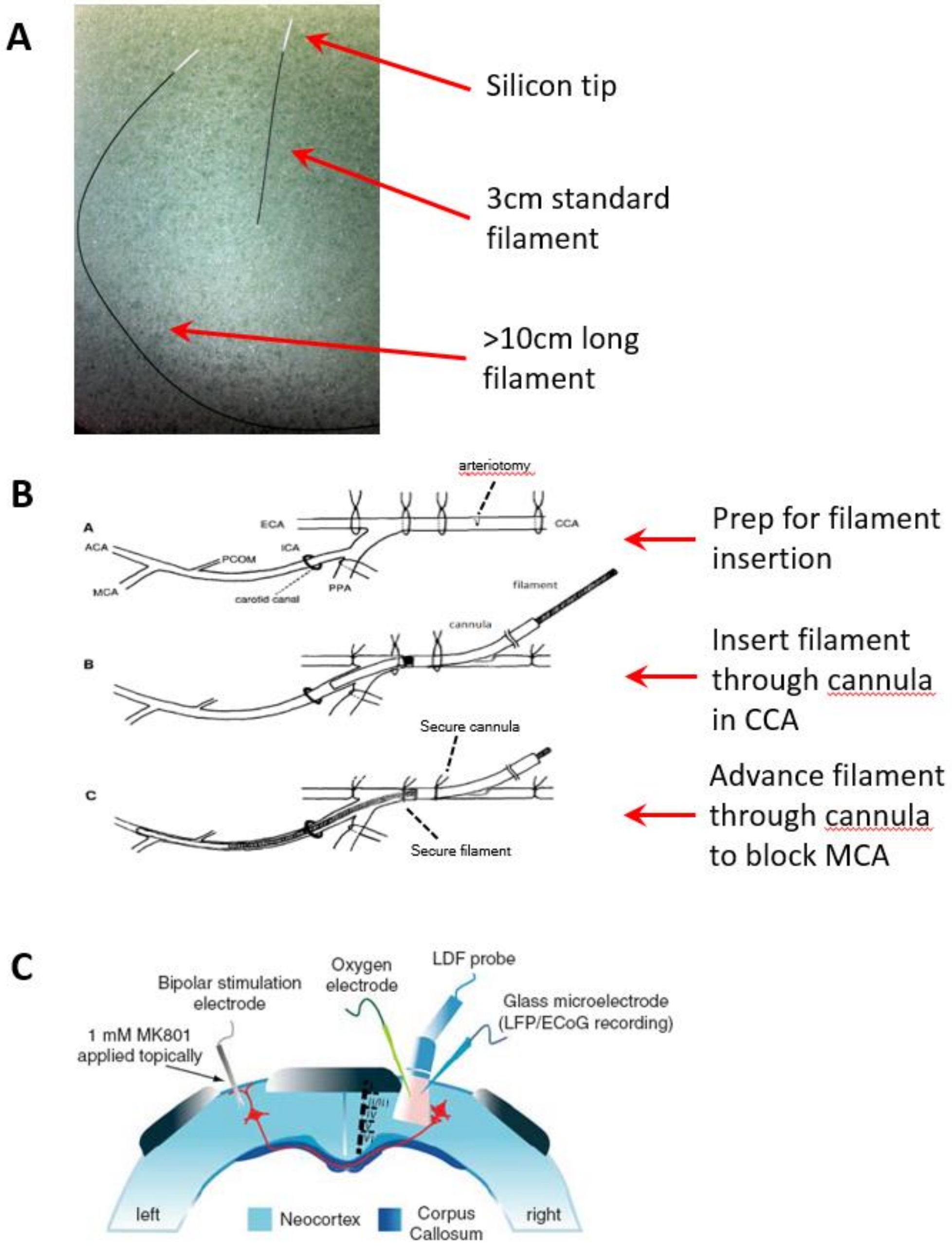

Supplementary Figure 2. Methodological techniques. A) The remote MCAO model utilised a custom-made 4-0 filament >10 cm long with a silicon tip (0.37 mm diameter, 5-6 mm length). The conventional MCAO model utilises the same filament but only 3 cm in length. All filaments were supplied by Doccol Corporation (Sharon, MA, USA). **B)** The remote MCAO model required ligation of both the ECA and CCA and retraction of the PPA. Two loose sutures were placed over the CCA close to the bifurcation with the ECA and ICA. An arteriotomy was made in the CCA. A guide cannula was inserted into the CCA up to the first loose suture and then

tightened to secure. The filament was inserted through the cannula in the CCA arteriotomy and advanced beyond the PPA and secured with the second loose suture on the CCA. The rat was then moved to the stereotaxic frame, and the long filament was advanced to occlude the MCA. Adapted from Memezawa *et al*.[35] **C)** Transcallosal stimulation was carried out through bilateral craniotomies over the somatosensory cortex. A bipolar stimulation electrode stimulated neurons of the left cortex which signalled to the right cortex through the corpus callosum. Evoked cerebral blood flow was measured using LDF (or Laser Speckle Contrast Imaging), evoked tpO_2 was measured using an oxygen electrode, and neuronal activity was measured using a LFP microelectrode. For craniotomy studies, 1 mM MK801 (NMDA receptor antagonist) was applied topically to the left cortex to prevent seizure activity and cortical spreading depression occurring in that hemisphere. Adapted from Piilgaard and Lauritzen.[17] Abbreviations: ACA = anterior cerebral artery; CCA = common carotid artery; ECA = external carotid artery; ICA = internal carotid artery; MCA = middle cerebral artery; PCOM = posterior communicating artery; PPA = pterygopalatine artery; LDF = laser Doppler flowmetry; LFP = local field potential; ECoG = electrocorticography.

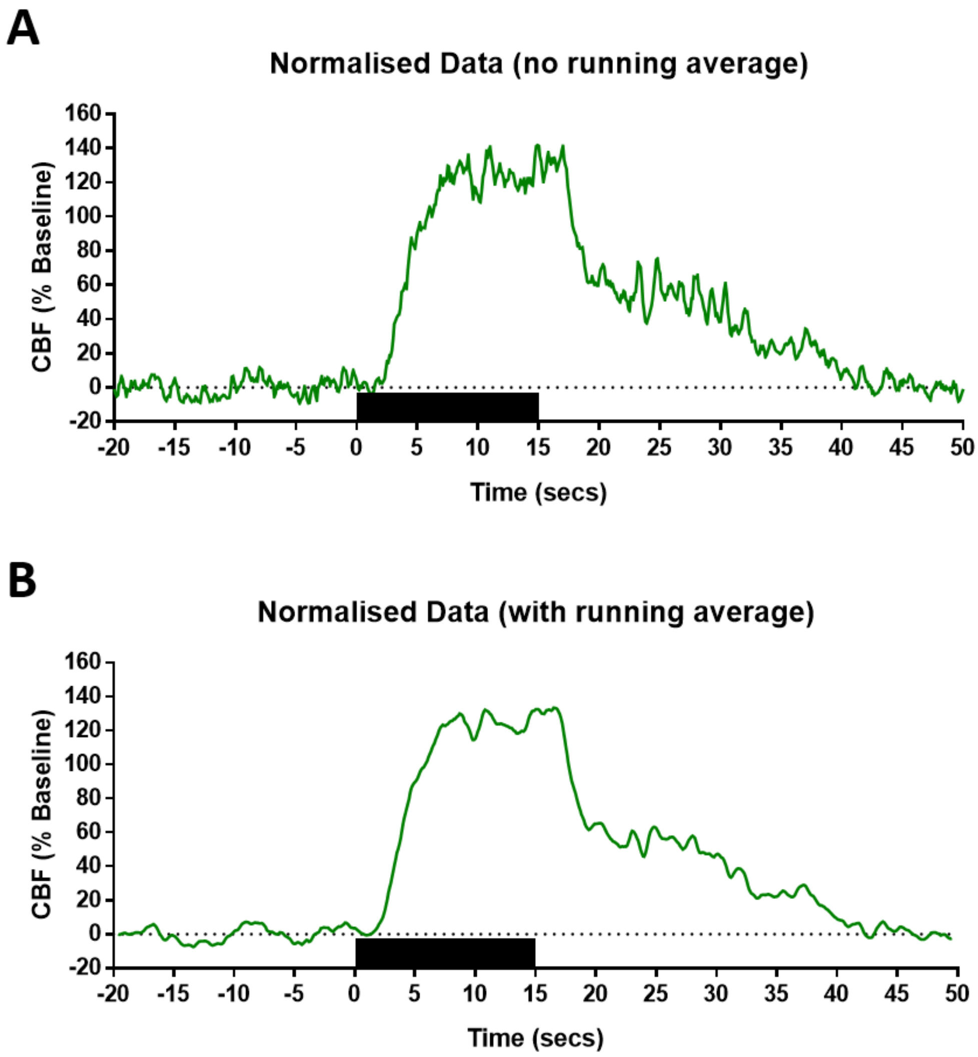

Supplementary Figure 3. Smoothing stochastic noise with a running average analysis method. A. Example evoked cerebral blood flow (CBF) in response to 10 Hz transcallosal stimulation for 15 secs. CBF during the stimulation was normalized to the CBF levels 20 secs prior to stimulation onset. CBF recordings were made at 0.1 sec intervals. The black rectangle indicates the stimulation period. **B.** The same evoked CBF response as (A) but calculated using a 1 sec running average to smooth the stochastic noise to perform maxima and area analysis in response to transcallosal stimulation.

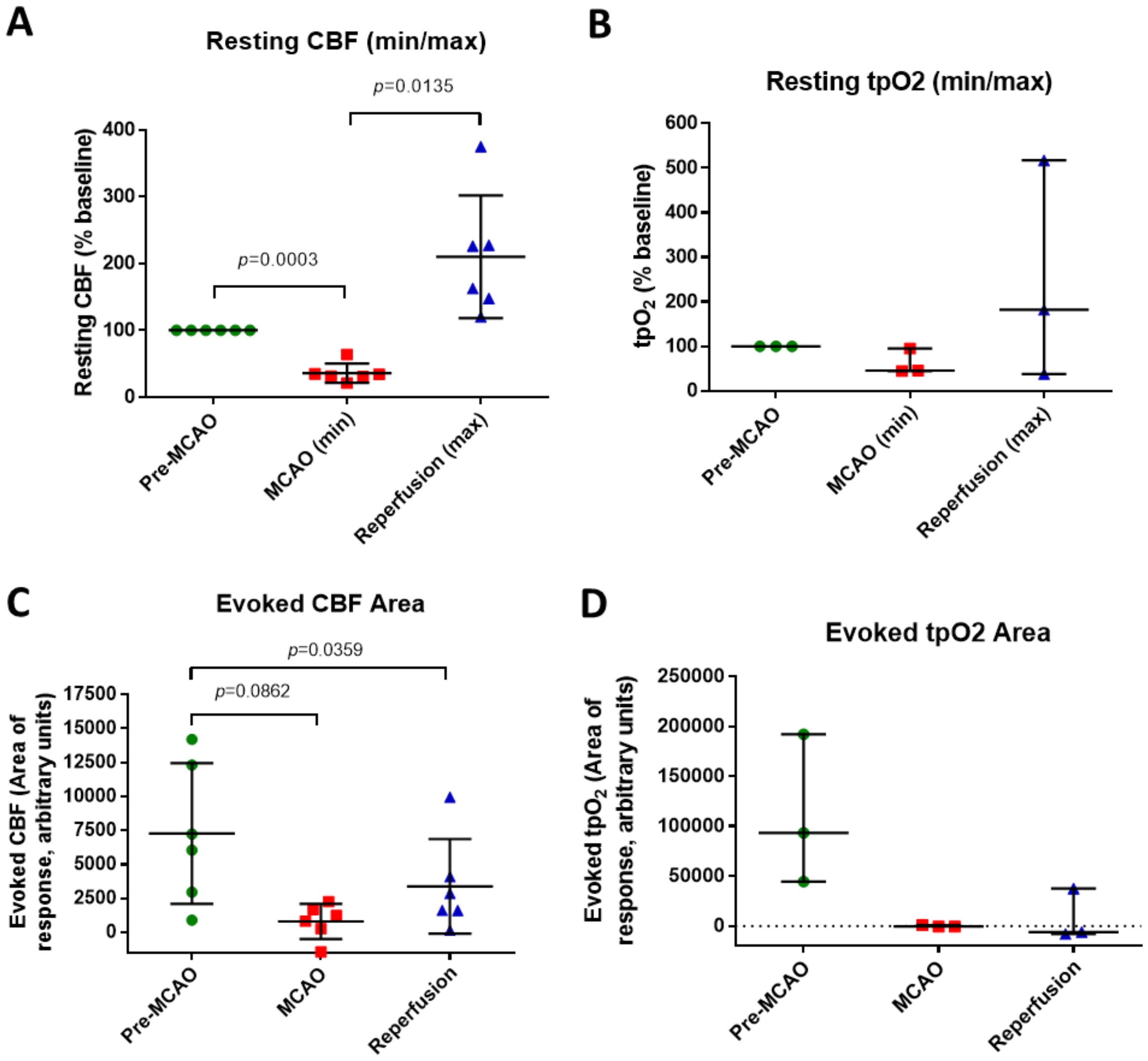

Supplementary Figure 4. Remote MCAO diminishes resting and evoked CBF and metabolism. A) Remote MCAO significantly reduced resting CBF (presented as minima) while reperfusion increased CBF (presented as maxima). **B)** Remote MCAO decreased resting tpO2 (minima) while reperfusion increased tpO2 (maxima). **C)** The area of evoked CBF in response to transcallosal stimulation was attenuated with remote MCAO and remained attenuated during reperfusion. **D)** The area of evoked tpO2 in response to transcallosal stimulation was reduced with remote MCAO and remained decreased during reperfusion. **A** & **C** had N = 6 per group and presented as mean ± SD. **B** & **D** had N = 3 per group and presented as median + range.

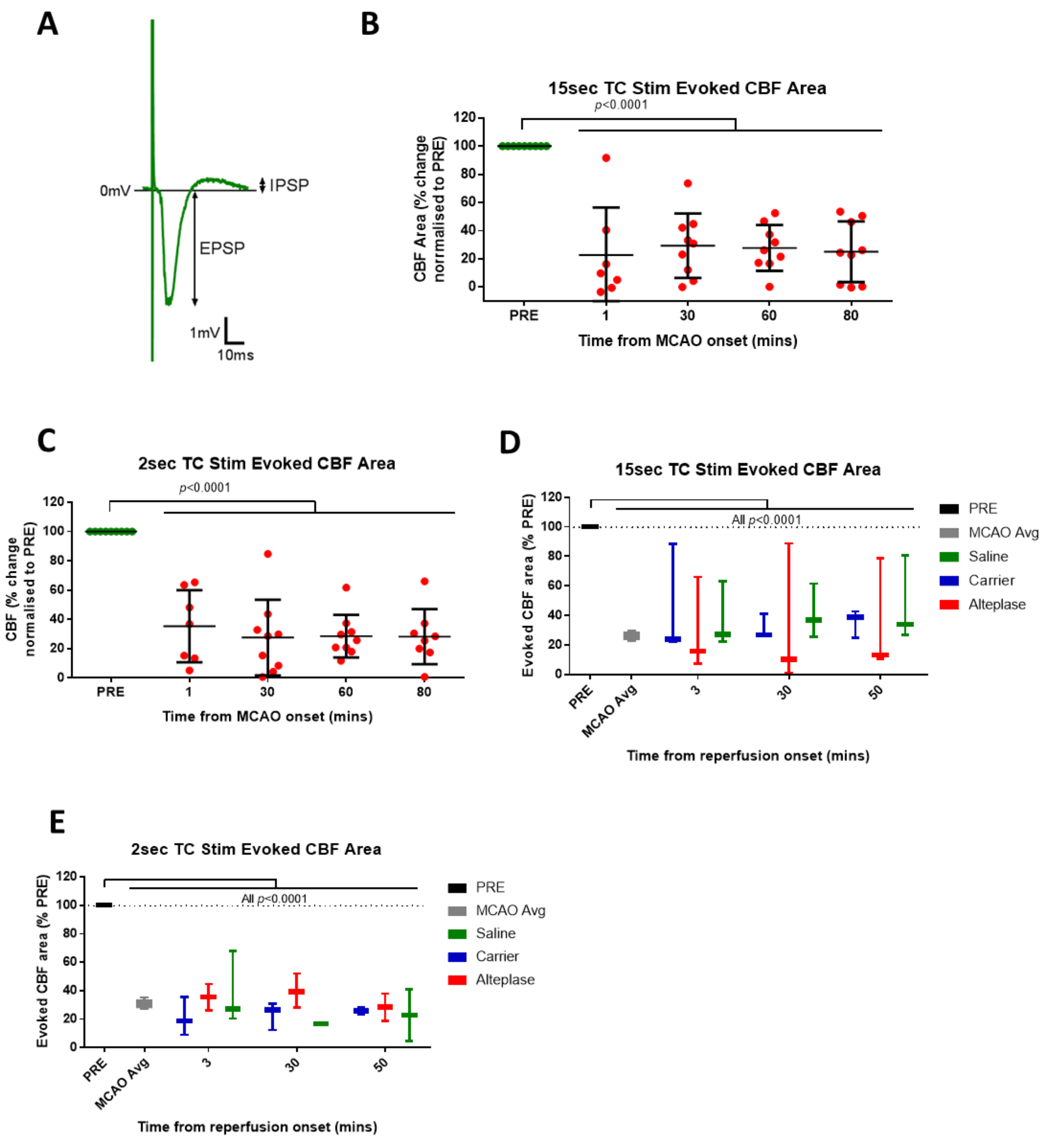

Supplementary Figure 5. Changes in the CBF response to transcallosal stimulation during remote MCAO and reperfusion. A) Representative local field potential trace, which was used to calculate both excitatory post-synaptic potential (EPSP) and inhibitory post-synaptic potential (IPSP). **B)** Evoked CBF area during 10 Hz transcallosal stimulation for 15 sec was significantly diminished during remote MCAO. **C)** Evoked CBF area during 10 Hz transcallosal

stimulation for 2 sec was significantly diminished during remote MCAO. **D)** Evoked CBF area during 10 Hz transcallosal stimulation for 15 sec during reperfusion following remote MCAO remained diminished and was not affected by alteplase. **E)** Evoked CBF area during 10 Hz transcallosal stimulation for 2 sec during reperfusion following remote MCAO remained attenuated and was not affected by alteplase. **B** & **C** had N = 9 per timepoint and presented as mean ± SD. **D** & **E** had N = 3 per group per timepoint and presented as median + range.

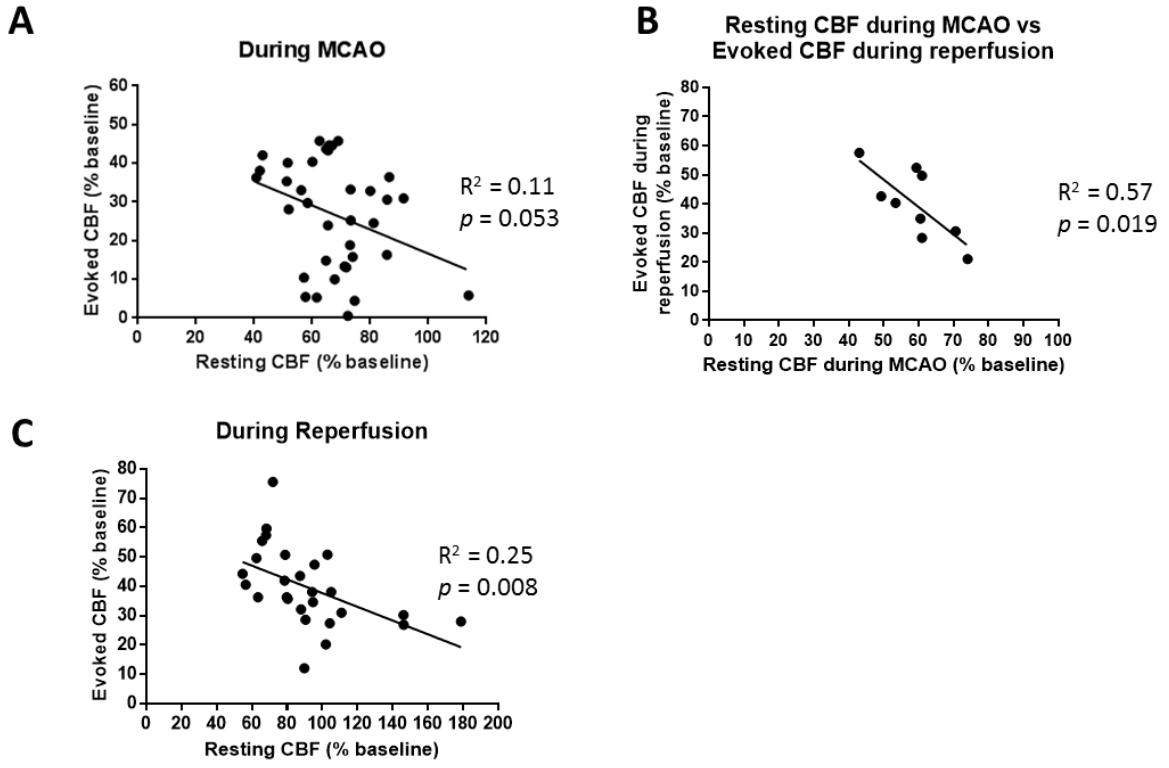

Supplementary Figure 6. Correlations between evoked CBF and resting CBF during remote MCAO and reperfusion. A) There was no significant correlation between the evoked CBF response and resting CBF level during MCAO. **B)** Decreased resting CBF during ischemia was correlated with increased evoked CBF during reperfusion. **C)** Decreased resting CBF during reperfusion was correlated to increased evoked CBF during reperfusion.

Supplementary Movies

Supplementary Movie 1. CBF decrease during remote MCAO. This movie shows a craniotomy over the right somatosensory cortex with CBF being recorded using Laser Speckle Contrast Imaging (Moor Instruments, Axminster, UK). At frame 412, remote MCAO occurred as indicated by a sharp drop in CBF, which rebounded but not to pre-MCAO levels.

Supplementary Movie 2. CBF restoration following filament retraction after remote MCAO. This movie shows a craniotomy over the right somatosensory cortex with CBF being recorded using Laser Speckle Contrast Imaging (Moor Instruments, Axminster, UK). At frame 214, remote reperfusion was induced as indicated by a sudden increase in CBF, followed by steadying of CBF.

Supplementary Movie 3. Evoked CBF increases in response to transcallosal stimulation. Two craniotomies with the somatosensory cortex of each hemisphere exposed. A stimulating electrode was placed in the rat's left cortex (right craniotomy in the movie). CBF was recorded using Laser Speckle Contrast Imaging (Moor Instruments, Axminster, UK). The movie, sped up 25x, shows three 15 sec stimulations of 10 Hz, 0.2 ms pulsewidth, 2 mA, 2 min inter-stimulation interval. The evoked CBF increase can be clearly observed in both cortices in response to stimulation.

Supplementary References

17. Piilgaard, H. & Lauritzen, M. Persistent increase in oxygen consumption and impaired neurovascular coupling after spreading depression in rat neocortex. J Cereb Blood Flow Metab 2009; 29: 1517-1527.

35. Memezawa, H., Minamisawa, H., Smith, M.L. & Siesjo, B.K. Ischemic penumbra in a model of reversible middle cerebral artery occlusion in the rat. Exp Brain Res 1992; 89: 67-78.
